# Adaptive Potential of Hybridization among Malaria Vectors: Introgression at the Immune Locus *TEP1* between *Anopheles coluzzii* and *A*. *gambiae* in ‘Far-West’ Africa

**DOI:** 10.1371/journal.pone.0127804

**Published:** 2015-06-05

**Authors:** Emiliano Mancini, Maria Ida Spinaci, Vasco Gordicho, Beniamino Caputo, Marco Pombi, José Luis Vicente, João Dinis, Amabélia Rodrigues, Vincenzo Petrarca, David Weetman, João Pinto, Alessandra della Torre

**Affiliations:** 1 Istituto Pasteur-Fondazione Cenci-Bolognetti, Dipartimento di Sanità Pubblica e Malattie Infettive, “Sapienza” Università di Roma, Rome, Italy; 2 UEI Parasitologia Médica, Instituto de Higiene e Medicina Tropical, Universidade Nova de Lisboa, Lisbon, Portugal; 3 Centro de Malária e outras Doenças Tropicais Instituto de Higiene e Medicina Tropical, Universidade Nova de Lisboa, Lisbon, Portugal; 4 Instituto Nacional de Saúde Pública., Bissau, Guinea Bissau; 5 Dipartimento di Biologia e Biotecnologie "C. Darwin", “Sapienza” Università di Roma, Rome, Italy; 6 Department of Vector Biology, Liverpool School of Tropical Medicine, Liverpool, United Kingdom; University of Crete, GREECE

## Abstract

“Far-West” Africa is known to be a secondary contact zone between the two major malaria vectors *Anopheles coluzzii* and *A*. *gambiae*. We investigated gene-flow and potentially adaptive introgression between these species along a west-to-east transect in Guinea Bissau, the putative core of this hybrid zone. To evaluate the extent and direction of gene flow, we genotyped site 702 in Intron-1 of the *para* Voltage-Gated Sodium Channel gene, a species-diagnostic nucleotide position throughout most of *A*. *coluzzii* and *A*. *gambiae* sympatric range. We also analyzed polymorphism in the thioester-binding domain (TED) of the innate immunity-linked thioester-containing protein 1 (*TEP1*) to investigate whether elevated hybridization might facilitate the exchange of variants linked to adaptive immunity and *Plasmodium* refractoriness. Our results confirm asymmetric introgression of genetic material from *A*. *coluzzii* to *A*. *gambiae* and disruption of linkage between the centromeric "genomic islands" of inter-specific divergence. We report that *A*. *gambiae* from the Guinean hybrid zone possesses an introgressed *TEP1* resistant allelic class, found exclusively in *A*. *coluzzii* elsewhere and apparently swept to fixation in West Africa (i.e. Mali and Burkina Faso). However, no detectable fixation of this allele was found in Guinea Bissau, which may suggest that ecological pressures driving segregation between the two species in larval habitats in this region may be different from those experienced in northern and more arid parts of the species’ range. Finally, our results also suggest a genetic subdivision between coastal and inland *A*. *gambiae* Guinean populations and provide clues on the importance of ecological factors in intra-specific differentiation processes.

## Introduction

*Anopheles gambiae* (Giles) and *A*. *coluzzii* (Coetzee & Wilkerson sp. n.) (formerly defined as *A*. *gambiae* s.s. M and S molecular forms based on X-linked SNPs in ribosomal DNA [1] are arguably the most important cryptic species of mosquitoes transmitting human malaria in sub-Saharan Africa. Restricted gene flow between *A*. *gambiae* and *A*. *coluzzii* in natural populations from sympatric areas of West and Central Africa is attributed to pre-mating mechanisms of reproductive isolation, selection against hybrids and ecologically-driven divergent selection [2, 3, 4, 5, 6]. Variation in larval habitats strongly influences species segregation: *A*. *gambiae* is associated with small ephemeral puddles, whereas *A*. *coluzzii* breeds in larger and more stable ponds, often created by agriculture, urbanization, or other human activities [7, 8, 9].

Genetic divergence between *A*. *gambiae* and *A*. *coluzzii* appears to be concentrated in "genomic islands of divergence" located in peri-centromeric regions of chromosome-X, -2 and -3 [10, 11], but it is also detectable in other smaller areas across the genome, some outside of centromeres [12, 13, 14, 15, 16]. A comparative genome-wide scan identified a significant area of inter-specific divergence on chromosome-3L, including five known or suspected immune response genes [17]. Of these, the thioester-containing protein 1 (*TEP1*) encodes a complement-like opsonin, binding of which triggers killing of gram-negative bacteria and protozoa via phagocytosis [18]. *TEP1* is highly polymorphic [19] and shows amino acid substitutions in the thioester-binding domain (TED) associated with pathogen resistance phenotypes [18]. In fact, experimental infections demonstrated that laboratory-reared *A*. *gambiae* individuals homozygous or heterozygous for TEP1*R1 [18] and *TEP1r* ^*B*^ [17] alleles are significantly more resistant to *Plasmodium* and bacterial infections than mosquitoes carrying other *TEP1* alleles. In contrast individuals carrying TEP1*R2 [18] and *TEP1r* ^*A*^ [17] alleles show less resistant phenotypes. *TEP1* genotyping of natural populations indicates that *TEP1r* ^*B*^ is absent or very rare in *A*. *gambiae*, but is fixed in *A*. *coluzzii* from West Africa (i.e. Mali and Burkina Faso) [17]. Given the relatively low rates and intensities of natural *Plasmodium* infection in both mosquito species, it was speculated that the most likely source of pathogen-mediated selection for resistance came from larval habitat [17]. Specifically, the longer-lasting and more biotically diverse aquatic milieu exploited by *A*. *coluzzii*, presumably harboring richer pathogen populations than temporary breeding sites exploited by *A*. *gambiae*, would exert higher selective pressures on the immune system of the former species [17, 20].

Although recent analyses suggest that hybridization between *A*. *gambiae* and *A*. *coluzzii* may be more dynamic than previously appreciated [6], the “Far-West” African region likely represents the most stable hybridization zone. High frequencies of *A*. *gambiae* x *A*. *coluzzii* hybrids have been repeatedly recorded in The Gambia (up to 7%) [21] and Guinea Bissau (>20%) [22, 23] leading to the hypothesis that these “Far-West” areas of the species’ range may represent a secondary contact zone in which local ecological settings have significantly disrupted reproductive barriers [21].

Hybrid zones offer an excellent opportunity to examine the outcome of genetic exchange of traits responsible for species segregation and to identify possible changes in ecological conditions inducing relaxation of the reproductive isolation found elsewhere in the sympatric range of their distribution [24]. Data collected so far on the genetic background of parental and hybrid individuals from the *A*. *gambiae*/*A*. *coluzzii* secondary contact zone indicate a preferential acquisition by *A*. *gambiae* of *A*. *coluzzii* alleles suggesting asymmetric introgression from *A*. *coluzzii* to *A*. *gambiae* [6, 23, 25].

In this paper we surveyed parental and hybrid mosquitoes from a West-to-East transect in Guinea Bissau to investigate gene-flow and potentially adaptive introgression between *A*. *coluzzii* and *A*. *gambiae* in their secondary contact zone. First, we evaluated the extent and direction of gene flow, using the species-informative site 702 in Intron-1 (Int-1^702^) of the Voltage-Gated Sodium Channel (VGSC) gene, located within the chromosome-2L "genomic island". This site is characterized by species-specific alleles in West and Central Africa (*A*. *coluzzii* = Int-1^C^; *A*. *gambiae* = Int-1^T^), which define species-specific Intron-1 haplogroups in strong linkage disequilibrium with the species-diagnostic X-linked rDNA SNPs [26, 27]. Second, we investigated the effect of hybridization on the exchange of adaptive alleles in the secondary contact zone by analyzing polymorphism in the catalytic TED domain of the *TEP1* gene on chromosome-3.

## Materials and Methods

### Field collected samples and species identification

**Fig 1:**
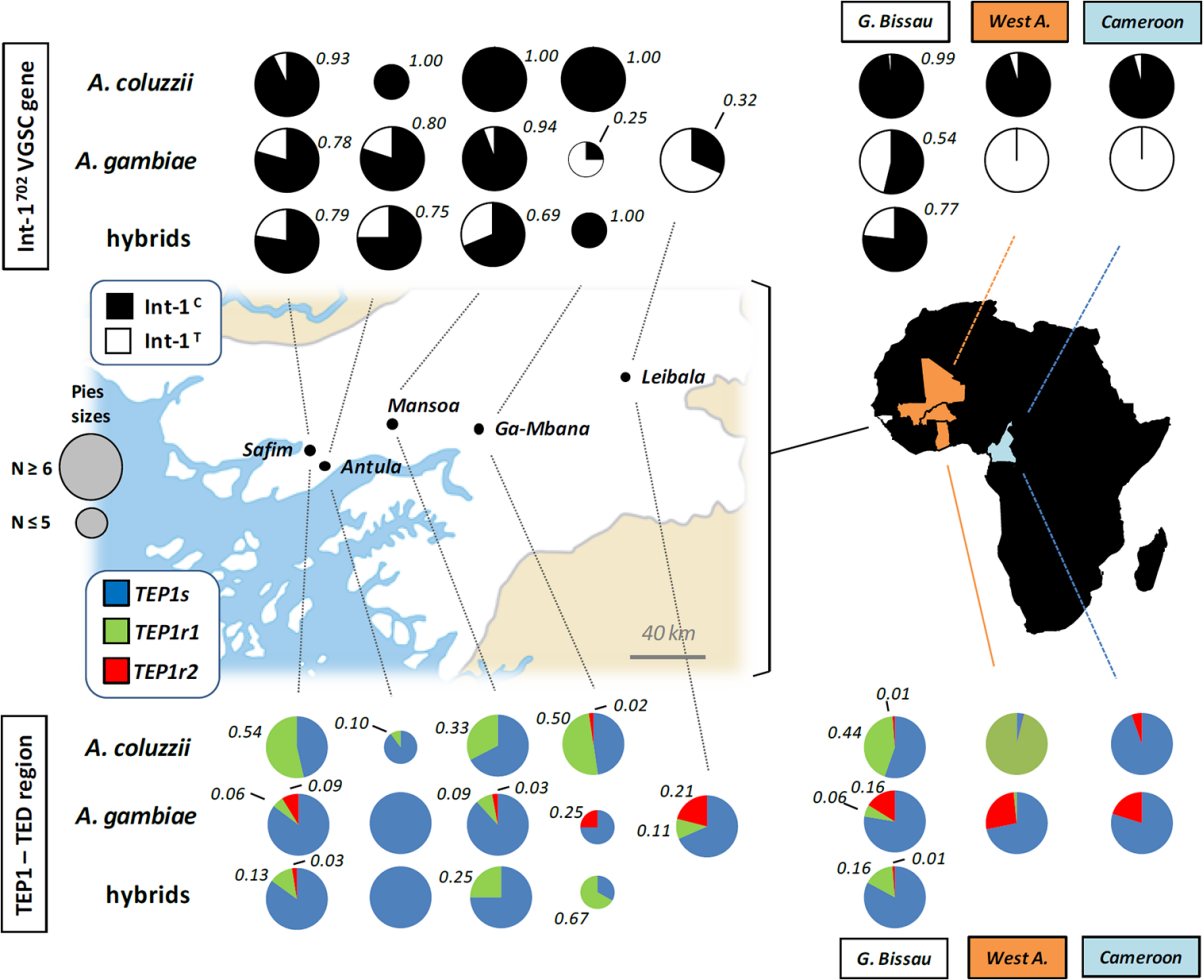
Int-1^702^ and *TEP1* allele frequencies along a west-to-east geographic transect in Guinea Bissau. Relative frequencies of Int-1^**702**^ VGSC (above) and *TEP1* (below) alleles are reported overall and in each of the five sampled villages for *A*. *coluzzii*, *A*. *gambiae* and hybrids. Numbers refer to relative frequencies of Int-1^**C**^ and of *TEP1r1* and *TEP1r2* alleles in Guinean sample. Overall allele frequencies in Guinea Bissau for both markers (this study) and from West-Africa and Cameroon (data from Gentile et al., 2004 and White et al., 2011) are reported on the right for comparison. GPS coordinates (UTM) of sampled Guinean villages are as follows: Antula (11° 53’ 49” N—15° 35’ 29” W), Safim (11° 57’ 00” N—15° 39’ 00” W), Mansoa (12° 04' 00'' N—15° 19' 00'' W), Ga-Mbana (12° 03' 00'' N—14° 55' 00'' W) and Leibala (11° 52' 53.96'' N—15° 37' 4.06'' W). Geographic map modified from Guinea Bissau sm03.png (Wikipedia image in public domain)

*Anopheles gambiae* s.l. adult females were selected from a larger sample collected in the rainy season of 2010 (October) in five villages located along a West-to-East geographical transect in Guinea Bissau: Antula (11° 53’ 49” N—15° 35’ 29” W), Safim (11° 57’ 00” N—15° 39’ 00” W), Mansoa (12° 04' 00'' N—15° 19' 00'' W), Ga-Mbana (12° 03' 00'' N—14° 55' 00'' W) and Leibala (11° 52' 53.96'' N—15° 37' 4.06'' W) (Fig 1). Field collections (not conducted in protected areas, nor involving endangered or protected species) were approved by and carried out under the guidance of the Guinea Bissau National Institute of Public Health (INASA). Indoor sampling was performed in private houses after permission by owners (informed on research aims) with CDC light traps in Antula and Ga-Mbana and with mechanical aspirators (Indoor Resting Collection, IRC) in Safim, Mansoa and Leibala.

Morphological identification was performed using the available taxonomic keys [28, 29, 30]. Specimens were stored in silica gel-filled tubes until DNA extraction using DNAzol (Life Technologies) or DNeasy Blood & Tissue Kit (Qiagen) was carried out. Identification of *A*. *gambiae* s.s. and *A*. *coluzzii* was carried out using two methods: *SINE*-PCR based on *SINE* insertion [31] and IMP-PCR based on IGS mutations [32]. We chose samples to be genotyped from within each sample site to increase the number of specimens of the less-frequent species and of the hybrid category. The latter includes all individuals heterozygous for both diagnostic markers (i.e. MS^SINE^/MS^IGS^, N = 31) and specimens showing discordant *SINE* and IGS PCR patterns (i.e. MM^SINE^/MS^IGS^, N = 4; SS^SINE^/MS^IGS^, N = 4; MS^SINE^/MM^IGS^, N = 2; from Safim and Mansoa), interpreted as being advanced generation hybrids (see [6, 11, 33, 34] for further details on species and hybrid identification in the secondary contact area).

### Genotyping of Int-1^702^ SNP

We genotyped Int-1^702^ using a primer-introduced restriction analysis assay (PIRA-PCR) designed on available Int-1 alignments [26, 27]. A forward primer, INTeco-f (5'-ATTATGCTCTTTACAATGCCAACGgAAT-3'), was designed to incorporate a C-to-G mismatch at the 4^th^ base from the 3'-end. In the presence of a ‘T’ at site 702 and of a fixed ‘C’ at site 703 (as observed in West Africa [26, 27 35]), the -3’ gAAT sequence of INTeco-f creates a recognition site for the *EcoRI* restriction enzyme (*i*.*e*. G’AATTC) within the PCR product amplified with INTeco-f and reverse primer INTa-r (5’-GGAATCTATCCACATTATCTG-3’). The restriction produces a 265 bp or a 240 bp band for Int-1^C/C^ and Int-1^T/T^ homozygotes, respectively; heterozygotes display both bands. PCR reactions were carried out in a 10 μl reaction which contained 1x Buffer, 1 pmol of each primer, 0.2 mM of each dNTP, 1.5 mM MgCl_2_, 2.5 U Taq DNA polymerase, and 8–10 ng of template DNA extracted from a single mosquito. Thermocycler conditions were 94°C for 10 min followed by thirty-five cycles of 94°C for 30 sec, 54°C for 30 sec and 72°C for 1 min, with a final elongation at 72°C for 10 min. Five microliters of each PCR product were digested with 10 U of EcoRI enzyme (New England Biolabs, UK) with 1× buffer in a final volume of 15 μl incubated at 37°C for 1 hour. The restriction products were run on 3% agarose gels stained with ethidium bromide. To obtain stronger bands on templates that proved difficult, a semi-nested PCR protocol was employed: on a first round of amplification, PCR products were obtained using INTeco-f and INTb-r (5’-ATCTTGGCAGATCATGGTCGG-3’), then diluted 1:100 and used as template for a second round of amplification under the PCR conditions described above.

### *TEP1* amplification and sequencing

A 456 bp fragment of the *TEP1*-TED domain was amplified with primers OB2996F (5'-CACGGTCATCAAGAACCTGGAC-3') [19] and EMTep1R (5’-TCCAGCAATGCCATCAACACAT-3'), the latter specifically designed for the aim of this work in order to avoid co-amplification of other *TEP*-related paralogs, which were instead pervasively co-amplified with other primer couples available in literature. Amplifications were performed in a 15 μl reaction-mix using 0.5–1.0 μl of template DNA using the High Fidelity AccuPrime Taq DNA Polymerase kit (Life Technologies) following manufacturer's guidelines. Thermocycler conditions were as follows: initial denaturation at 94°C for 2 min followed by 35 cycles of 94°C for 30 sec, 54°C for 30 sec, 68°C for 1 min, with a final elongation at 68°C for 7 min. The resulting products were analysed on 1–2% agarose gels stained with GelRed (Biotium), purified with the SureClean Kit (Bioline) and sequenced at the BMR Genomics s.r.l. (Padua, Italy). Sequences are available in GenBank under Accession Numbers KR091079—KR091309.

### Sequence and population genetic analysis

*TEP1* chromatograms were edited and trimmed to remove low-quality base calls with Staden Package ver. 2003.1.6 [36]. A final 387 bp alignment of genotype sequences was produced using MAFFT ver. 7 [37] and alleles phased using the PHASE algorithm [38]. Resulting *TEP1* alleles were identified as susceptible or resistant based on key residues in the catalytic loop and pre-α- loop found in the *TED* portion [39]. The sequenced *TED* fragment did not allow us to discriminate among previously described variants within susceptible and resistant *TEP1* allelic classes, because such distinction is also based on residues outside the catalytic and pre-α- loop region [17, 18]. So, for the purpose of this study, we chose to name allelic classes as follows: *TEP1s* = 'susceptible' class, comparable to TEP1*S1, TEP1*S2, TEP1*S3 [18] and *TEP1s* [17]; *TEP1r1* = 'resistant' allele comparable to TEP1*R1 [18] and *TEP1r* ^*B*^ [17]; *TEP1r2* = *TEP1* 'resistant' allele plausibly corresponding to TEP1*R2 [18] and *TEP1r* ^*A*^ [17]. A median-joining network was built for *TEP1s* and *TEP1r* allelic classes with NETWORK ver. 4.510 [40]. *TEP1* genotyping data obtained by B. White and collaborators [17] were used for comparison.

DnaSP v5.10.1 [41] was used to estimate genetic polymorphism and to perform neutrality tests, *i*.*e*. Tajima's D, Fu & Li D* and Wall's Q statistics. *F*-statistics (*F*_*ST*_ and *F*_*IS*_) [42], departures from Hardy-Weinberg Equilibrium (HWE) and linkage disequilibrium (LD) were estimated for *TEP1* and Int-1^702^ genotyping data using ARLEQUIN 3.5 [43] and GENEPOP ‘007 [44].

## Results and Discussion

The westernmost extreme of the sympatric range of *Anopheles coluzzii* and *A*. *gambiae* is believed to be the core of a secondary contact region where disruption of genetic association is observed among "genomic islands" of divergence on centromeres of chromosome-X, -2 and -3, and a preferential transfer of genetic material from *A*. *coluzzii* to *A*. *gambiae* occurs (i.e., asymmetric introgression) [21, 23, 25, 34]. These phenomena are confirmed by our results from the genotyping of the Int-1^702^ SNP of the VGSC gene in Guinean populations (Fig 1). Results show that, as in the rest of the species range, Int-1^C^ allele is almost fixed in Guinean *A*. *coluzzii*. However, in contrast to other areas, it is also found at high frequencies (up to 94%) in sympatric *A*. *gambiae* populations from the coastal/cropland areas of Antula, Safim and Mansoa. Thus, the association between Int-1^702^ and species-specific markers on chromosome-X (i.e. IGS, *SINE*) observed elsewhere [26, 27] is lost in these populations (S1 Table). It is worth noting that the very low frequency of *kdr*-associated mutations in these three *A*. *gambiae* sample sites (Vicente JL, personal communication) precludes the explanation that this unprecedented Int-1^702^ pattern might result from hitchhiking driven by insecticide selective pressure acting on *kdr* locus, as shown to occur in other African regions [35, 45].

**Fig 2:**
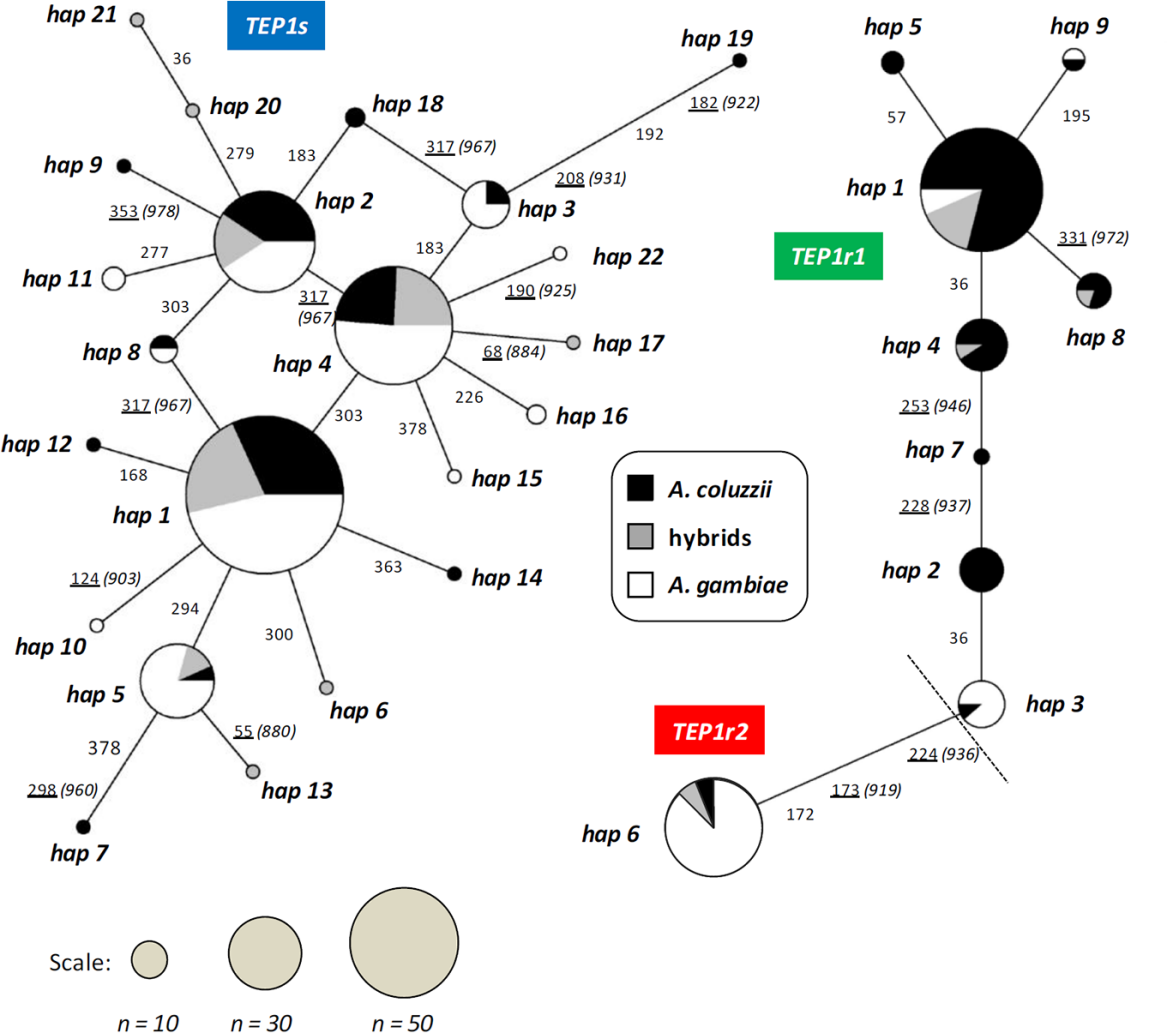
*TEP1* median-joining networks depicting relationships among *TEP1s* (left) and *TEP1r* (right) related haplotypes. Haplotype frequencies are proportional to pie sizes. Site numbering of synonymous and non-synonymous (underlined) mutations along branches follows our 387 bp *TEP1* alignment, those of replaced amino acids (in brackets) follows Blandin et al., 2009. *TEP1s* and *TEP1r* networks are separated by a total of 32 fixed mutations, 12 at synonymous and 20 non-synonymous sites, respectively.

Previously, no data were available from the “Far-West” secondary contact region on functional polymorphisms of potential adaptive significance (such as in immune-related genes) that differentiate *A*. *coluzzii* and *A*. *gambiae* in the rest of their sympatric range. Our genetic analysis of *TEP1*-TED—whose *TEP1r1* allele confers high resistance to pathogens and is confined to *A*. *coluzzii* in West and Central Africa [17] (Fig 1)—provides the first insights on the exchange and polymorphism of this potentially adaptive protein in the “Far-West” region. First, susceptible (*TEP1s*) and resistant (*TEP1r1* and *TEP1r2*) alleles are shown to be shared by the two species (Fig 1). Indeed, the occurrence of the *TEP1r1* allele in *A*. *gambiae* at frequencies up to 11% contrasts with the virtual absence of this allele in the rest of the species range. Lack of *TEP1r1* private haplotypes in Guinean *A*. *gambiae* suggest that this allele was acquired from *A*. *coluzzii* (Fig 2).

**Fig 3:**
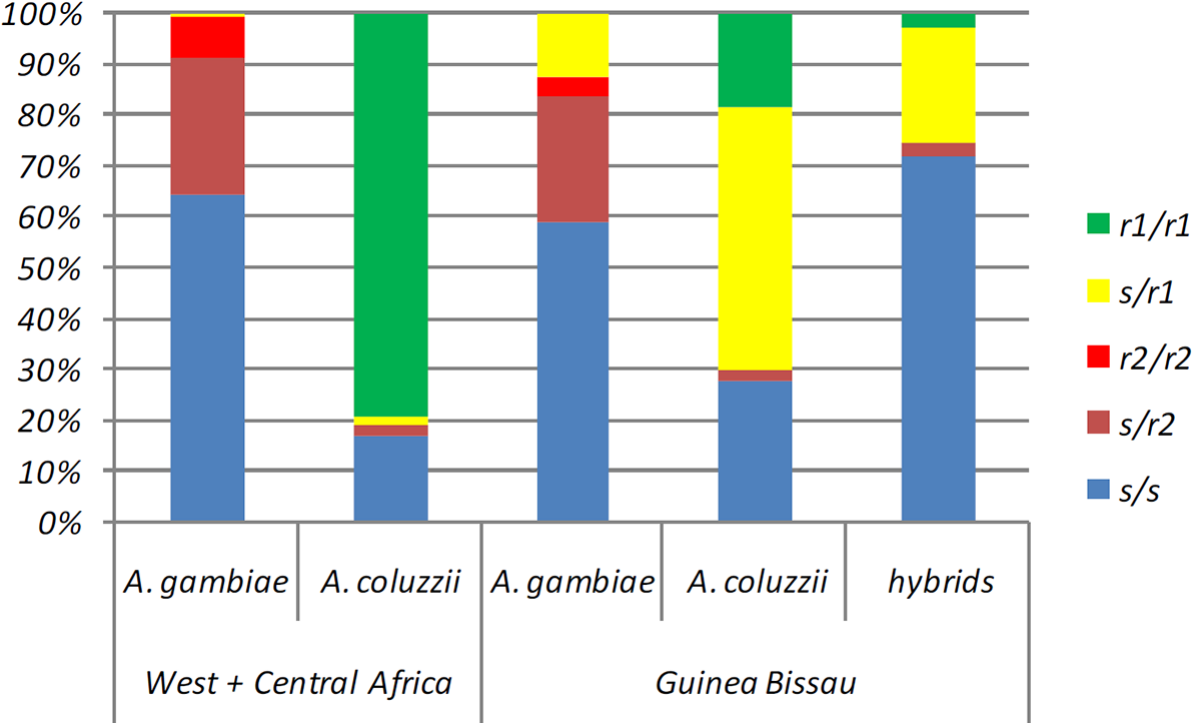
Comparison of *TEP1* genotype distribution in West/Central Africa and Guinea Bissau. Relative frequencies of *TEP1* genotypes in Guinea Bissau are reported in Table 1 and those from West and Central Africa are from White et al. (2011).

Yet, despite evidence of inter-specific gene-flow, significant genetic differentiation between *A*. *coluzzii* and *A*. *gambiae* remains (*F*_*ST*_ = 0.19, S2 Table), probably reflecting past segregation of *TEP1* resistant alleles in the hybridizing parental species. *TEP1r1* is more frequent in *A*. *coluzzii* than in *A*. *gambiae* (*χ* ^*2*^ = 46.9; *p*<0.001), whereas the opposite is observed for *TEP1r2* (*χ* ^*2*^ = 20.8; *p*<0.001) (Fig 1, Table 1). Second, our data highlights that *TEP1r1* allele frequency varies from 0.1% to 0.5% in Guinean *A*. *coluzzii* but never reach fixation (Table 2, Fig 3) as it occurs in co-specific populations from northern savanna areas of West Africa [17].

**Table 1:** Int-1^702^ and *TEP1* genotype frequencies and *F*_*IS*_.

			*Int-1* ^*702*^ *genotype frequencies (%)*				*TEP1 genotype frequencies (%)*					
		N	*Int-1* ^*C/C*^	*Int-1* ^*C/T*^	*Int-1* ^*T/T*^	*F*_*IS*_	*s/s*	*s/r1*	*r1/r1*	*s/r2*	*r2/r2*	*F*_*IS*_
**Overall**	***A*. *coluzzii***	85	97.7	2.3	-	-0.01	28.2	51.8	17.7	2.3	-	-0.07
	**hybrids**	41	65.8	22.0	12.2	**0.39** *	68.3	26.9	2.4	2.4	-	-0.01
	***A*. *gambiae***	105	43.8	20.0	36.2	**0.60** **	59.0	12.4	-	24.8	3.8	-0.01
	**Total**	231	67.5	13.9	18.6	**0.64** **	49.3	29.4	6.9	12.6	1.7	**0.07** **
**Antula**	***A*. *coluzzii***	5	100.0	-	-	n.a.	80.0	20.0	-	-	-	n.a.
	**hybrids**	10	70.0	10.0	20.0	**0.76** *	100.0	-	-	-	-	n.a.
	***A*. *gambiae***	10	60.0	40.0	-	-0.20	100.0	-	-	-	-	n.a.
**Safim**	***A*. *coluzzii***	14	85.7	14.3	-	-0.04	21.4	50.0	28.6	-	-	-0.04
	**hybrids**	20	60.0	35.0	5.0	0.02	70.0	25.0	-	5.0	-	-0.12
	***A*. *gambiae***	17	64.7	29.4	5.9	0.13	70.6	11.8	-	17.6	-	-0.10
**Mansoa**	***A*. *coluzzii***	23	100.0	0.0	-	n.a.	34.8	65.2	-	-	-	**-0.47** *
	**hybrids**	8	62.5	12.5	25.0	0.74	50.0	50.0	-	-	-	-0.27
	***A*. *gambiae***	17	94.1	-	5.9	**1.00** **	76.5	17.6	-	5.9	-	-0.08
**Ga-Mbana**	***A*. *coluzzii***	43	100.0	-	-	n.a.	20.9	48.8	25.6	4.6	-	-0.01
	**hybrids**	3	100.0	-	-	n.a.	-	66.7	33.3	-	-	-0.33
	***A*. *gambiae***	4	25.0	-	75.0	1.00	50.0	-	-	50.0	-	-0.20
**Leibala**	***A*. *gambiae***	57	21.1	21.1	57.8	**0.52** **	43.9	14.0	-	35.1	7.0	-0.04

Genotype frequencies of Int-1^702^ and *TEP1* in *A*. *gambiae* s.s. (i.e. SS^SINE^/SS^IGS^), *A*. *coluzzii* (i.e. MM^SINE^/MM^IGS^) and hybrids (i.e. 31 MS^SINE^/MS^IGS^, 4 MM^SINE^/MS^IGS^, 4 SS^SINE^/MS^IGS^ and 2 MS^SINE^/MM^IGS^) are reported as percentages (%). N = sample size, *F*_*IS*_ = inbreeding coefficient. Significant *F*_*IS*_ indicating deviations from HWE are reported in bold:

*p< 0.05

**p<0.001.

**Table 2:** *TEP1* polymorphism and neutrality tests.

		n	H	Hd	S	Eta	π	θ	N-SYN	SYN	π_A_	π_S_	D^syn^	D*	Q
**By allelic classes *(all)***	***TEP1s***	325	22	0.75	21	21	0.3	0.9	9	12	0.1	1.0	-1.27	**-5.16** *	0.09
	***TEP1r1***	100	8	0.59	6	6	0.3	0.0	3	3	0.2	0.5	-0.55	1.12	0.00
	***TEP1r2***	37	1	-	-	-	-	-	-	-	-	-	n.a.	n.a.	n.a.
***TEP1s (by groups)***	***A*. *coluzzii***	94	12	0.73	12	12	0.3	0.6	5	7	0.2	0.9	-1.03	**-3.49** *	0.17
	***A*. *gambiae***	163	11	0.77	9	9	0.3	0.4	3	6	0.1	1.1	-0.27	-1.13	0.00
	**hybrids**	68	9	0.72	8	8	0.3	0.4	3	5	0.1	0.9	-0.62	-1.75	0.00
***TEP1r1 (by groups)***	***A*. *coluzzii***	74	8	0.56	6	6	0.3	0.3	3	3	0.2	0.5	-0.50	0.23	0.00
	***A*. *gambiae***	13	3	0.56	3	3	0.3	0.2	2	1	0.3	0.2	-1.15	0.05	0.67
	**hybrids**	13	3	0.41	2	2	0.1	0.2	1	1	0.1	0.3	-0.27	-0.41	0.00
**In each locality *(all allelic classes)***	**Antula**	50	8	0.69	46	46	0.7	2.7	*n*.*a*.	*n*.*a*.	0.4	1.7	**-2.15** *	**-5.51** *	**0.76** ***
	**Safim**	102	16	0.85	52	52	4.4	2.6	*n*.*a*.	*n*.*a*.	3.3	8.3	**2.34** *	1.11	**0.50** ***
	**Mansoa**	96	16	0.79	50	51	4.3	2.5	*n*.*a*.	*n*.*a*.	3.3	7.8	1.67	1.06	**0.53** **
	**Ga-Mbana**	100	14	0.80	48	48	5.7	5.7	*n*.*a*.	*n*.*a*.	4.3	10.2	**3.49** ***	**1.69** *	**0.58** ***
	**Leibala**	114	10	0.83	45	45	4.5	4.5	*n*.*a*.	*n*.*a*.	3.2	9.0	**3.09** **	**1.91** *	**0.67** ***
**Overall**		*462*	*31*	*0*.*85*	*60*	*61*	*4*.*7*	*2*.*3*	*n*.*a*.	*n*.*a*.	*3*.*5*	*8*.*8*	***2*.*61*** *	*-0*.*58*	***0*.*34*** ***

Summary statistics for *TEP1* sequence polymorphism are reported a) within each *TEP1* allelic class in the whole sample, b) within *TEP1s* and *TEP1r1* in *A*. *gambiae*, *A*. *coluzzii* and hybrids, c) for the whole Guinean sample and within each locality (all *TEP1* allelic classes). n = n° of alleles, H = n° of haplotypes, Hd = haplotype diversity, S = n° of segregating sites, Eta = total n° of mutations, π = nucleotide diversity (at non-synonymous = π_a_ or at synonymous = π_s_ sites), θ = Watterson's estimator, N-SYN = n° of non-synonymous mutations, SYN = n° synonymous mutations, Tajima D^syn^ = Tajima's *D* test based on synonymous substitutions only. *n*.*a*. = not applicable. Deviations from neutrality tests are in bold:

* *p*< 0.05

** *p*< 0.01

*** *p*< 0.001.

**Fig 4:**
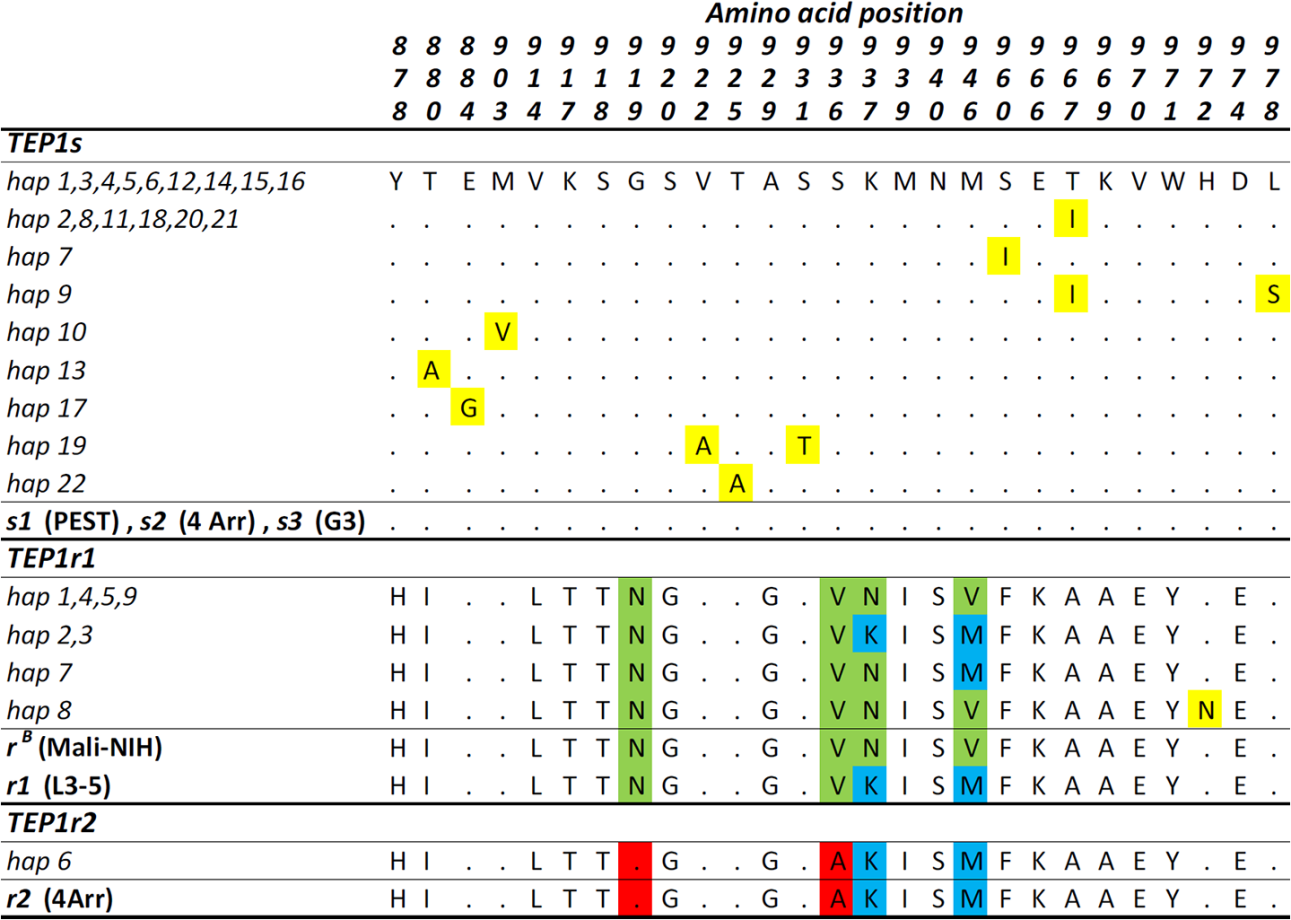
Amino acid variability in *TEP1*-TED region in Guinea Bissau. Protein alignment showing variable positions in the 387 bp of TED region of *TEP1* analyzed is shown. Positions are numbered following Blandin et al., 2009. Shading highlights amino acid differences within and between *TEP1s*, *TEP1r1* and *TEP1r2* allelic classes. *TEP1* allele designations refer to sequences from laboratory strains (Mali L3-5, Mali-NIH, PEST, 4Arr, and G3). *TEP1* haplotypes (based on nucleotide sequences and named as in Fig 2) reported in the left side of each row code for the same protein variant.

Polymorphism analysis shows that the *TEP1*-TED portion analyzed is well-conserved within each allelic class (*TEP1s*: π_S_ = 1.0, π_A_ = 0.1; *TEP1r1*: π_S_ = 0.5, π_A_ = 0.2; Table 2), likely retaining functionally-relevant residues known to confer resistance or susceptibility to pathogens [18, 39] (Fig 4). The four most common *TEP1s* haplotypes (-hap1,-hap2,-hap4 and-hap5) are those found in the rest of Africa [17, 19] and are shared between species (Fig 2). Of the resistant alleles (Figs 2 and 4), the most frequent *TEP1r1* haplotype found is related to a functional variant found exclusively in West-Africa (*i*.*e*. *TEP1r* ^*B*^) [17], and a single *TEP1r2* haplotype is observed along the entire transect, corresponding to Tep1*R2 [18] and *TEP1r* ^*A*^, which has a broad distribution [17]. Some novel *TEP1r1* protein variants (i.e.-hap7,-hap8) are also observed; the phenotypic consequences of these are currently unknown.

Finally, our analysis suggests intra-specific genetic subdivision within *A*. *gambiae* from coastal areas of Guinea Bissau eastwards (Fig 1). Firstly, *A*. *gambiae* populations from Western coastal/flooded cropland areas (i.e. Safim, Antula and Mansoa) show higher frequencies of Int-1^C^ (mean = 85.2%) than those from drier savanna-like areas in the eastern part of the geographical transect (i.e. Ga-Mbana and Leibala; mean Int-1^C^ frequency = 29.2%) (*χ* ^*2*^ = 20.1; *p*<<0.001). These coastal *A*. *gambiae* populations are also significantly differentiated from the easternmost population examined for the *TEP1* locus (i.e. Leibala; *F*_*ST*_ = 0.34–0.45, *p*<0.05; S2 Table). Moreover, *TEP1r2* frequency does not exceed 9.0% in the *A*. *gambiae* coastal populations, but is significantly higher (21%) in Leibala (*χ* ^*2*^ = 6.855; *p*<0.01) (Fig 1, Table 1). In addition, *A*. *gambiae* from Leibala also differs from coastal populations via near-exclusivity of the *TEP1r1*-hap3 haplotype (shared with only one *A*. *coluzzii* from Ga-Mbana, Fig 2), whose coding sequence contains two residues ('K' and 'M') related to the phenotypically-resistant TEP1*R1 [18], a possible recombinant *TEP1r* ^*B*^/*R2 allele [17] (Fig 4). Notably, this West-to-East genetic discontinuity is also revealed by the analysis of chromosome-3 microsatellite polymorphisms along the same geographic transect in Guinea Bissau (Pinto J., personal communication). This pattern may be due either to a different origin of “coastal” and “inland” *A*. *gambiae* populations, or to natural selection promoting niche partitioning and, hence, genetic splitting at a local scale within this species. It is worth noting that intra-specific genetic divergence between geographically close populations of *A*. *gambiae* belonging to different ecological settings has already been reported from the “Far-West” region (i.e. from The Gambia), where a potential role of introgressive hybridization in triggering this divergence at a meso-geographical scale has been hypothesized [46].

## Conclusions

The introgression of the *TEP1r1* allele from *A*. *coluzzii* to *A*. *gambiae* in Guinea Bissau (Fig 1) shows that hybridization can promote the transfer of potentially adaptive immune-resistant alleles from a 'donor' (*A*. *coluzzii*) to a 'recipient' (*A*. *gambiae*) vector species. Introgressive hybridization may favor the rapid acquisition of advantageous traits from one species to another, but the adaptive significance (or fitness effects) of the genetic variant entering the 'recipient' species should be ascertained [47]. It is tempting to speculate that the observed absence of fixation and/or relatively low frequency of the ‘novel’ *TEP1r1* acquired by *A*. *gambiae* in Guinea Bissau could be due to a lower adaptive benefit of this allele to the 'recipient' species in this region, where hybridization between *A*. *gambiae* and *A*. *coluzzii* is highest and stable. It would be interesting to monitor *TEP1* allele exchange also in other African regions where assortative mating was shown to occasionally break down [6] and to assess whether introgressed resistant-alleles increase in frequency in *A*. *gambiae*: this could enhance the immune responsiveness of this species and, thus, its ability to compete with *A*. *coluzzii* in permanent larval sites, with potential repercussions on vector ecology, distribution and, eventually, malaria transmission especially in those areas where larval habitats are strongly segregated. In fact, it has been hypothesized that the more biotically diverse aquatic milieu and the higher bacterial load in larval sites typical of *A*. *coluzzii* in dry savannah areas are the most likely selective forces driving fixation of *TEP1r1* in these northern populations [17, 20]. The observed lack of fixation of *TEP1r1* in Guinea Bissau might be related to a greater availability of water resources due to a relatively higher annual rainfall regime in this westernmost region when compared to northern savanna areas [48]. The more humid ecological context of Guinea Bissau is likely to cause frequent replenishment of *A*. *coluzzii* larval habitats with clean-water all-year round, thus diluting the bacterial load in permanent ponds and possibly reducing immune stress for mosquito larvae. Indeed, mean annual precipitation in Guinea Bissau (e.g. ~1500 mm/yr in Leibala to 2000 mm/yr in Antula) is considerably higher than in northern regions (e.g., Mali: Bamako, 1100 mm/yr; Burkina Faso: Ouagadougo, 800 mm/yr; Bobo Dioulasso, 1100 mm/yr) [25, 49]. The present data on *TEP1* allele distributions in Guinea Bissau could indirectly support the role of pathogens in potentially driving and maintaining fixation of *TEP1r1* in *A*. *coluzzii* larvae in Mali and Burkina Faso, where a lower and seasonal precipitation regime may increase water stagnation and concentration of organic matter in permanent breeding sites, thereby increasing pathogen density and diversity. This might also explain the lower frequency or absence of the *TEP1r1* allele in *A*. *coluzzii* collected close to coastal areas of Ghana and Cameroon [17]. Further studies testing correlations among *TEP1* genotype frequencies, chromosomal inversion polymorphisms (known to be highly differentiated between coastal Guinea Bissau and inland/northern savannah areas [50]), climatic/ecological conditions and pathogen loads in breeding sites are needed to confirm these hypotheses.

## Supporting Information

S1 Table Linkage disequilibrium (LD) between Int-1^702^ (2L chromosome) and IGS species-specific SNPs (X chromosome). LnLHood LD = likelihood of linkage disequilibrium; LnLHood LE = likelihood of linkage equilibrium; *p* (LD) = probabilities from likelihood ratio tests (significant LD are in bold).
(DOC)

S2 Table Pairwise comparisons of F_*ST*_ based on *TEP1* (above diagonal) and Int-1^702^ (below diagonal) allele frequencies. Pairwise comparisons of F_*ST*_ between species (co. = *A*. *coluzzii*, ga = *A*. *gambiae*) and hybrids (= hyb.) are reported either overall in Guinea Bissau, or within/among populations. Significance of F_*ST*_ was assessed by performing 500 replicates with a non-parametric permutation test; significant *p*<0.05 are in bold.
(DOC)
