# Supplementary material for: Adaptive Potential of Hybridization among Malaria Vectors: Introgression at the Immune Locus TEP1 between Anopheles coluzzii and A. gambiae in ‘Far-West’ Africa
Source: PLoS One. 2015 Jun 5;10(6):e0127804. doi: 10.1371/journal.pone.0127804 (PMC4457524; doi:10.1371/journal.pone.0127804)
Supplement: S1 Table — LnLHood LD = likelihood of linkage disequilibrium; LnLHood LE = likelihood of linkage equilibrium; p (LD) = probabilities from likelihood ratio tests (significant LD are in bold). (DOC) [file pone.0127804.s001.doc]

**S1. Linkage disequilibrium (LD) between Int-1^702^ (2L chromosome) and IGS species-specific SNPs (X chromosome).**
