# Supplementary material for: Adaptive Potential of Hybridization among Malaria Vectors: Introgression at the Immune Locus TEP1 between Anopheles coluzzii and A. gambiae in ‘Far-West’ Africa
Source: PLoS One. 2015 Jun 5;10(6):e0127804. doi: 10.1371/journal.pone.0127804 (PMC4457524; doi:10.1371/journal.pone.0127804)
Supplement: S2 Table — Pairwise comparisons of FST between species (co. = A. coluzzii, ga = A. gambiae) and hybrids (= hyb.) are reported either overall in Guinea Bissau, or within/among populations. Significance of FST was assessed by performing 500 replicates with a non-parametric permutation test; significant p<0.05 are in bold. (DOC) [file pone.0127804.s002.doc]

**S2. Pairwise comparisons of F*_ST_* based on *TEP1* (above diagonal) and Int-1^702^ (below diagonal) allele frequencies.**
